# Clinical Outcomes of Repeated Intraventricular Transplantation of Autologous Bone Marrow Mesenchymal Stem Cells in Chronic Haemorrhagic Stroke. A One-Year Follow Up

**DOI:** 10.2174/1874205X01711010074

**Published:** 2017-12-19

**Authors:** Asra Al Fauzi, Purwati Sumorejo, Nur Setiawan Suroto, Muhammad Arifin Parenrengi, Joni Wahyuhadi, Agus Turchan, Ferdiansyah Mahyudin, Heri Suroto, Fedik Abdul Rantam, Mochammad Hasan Machfoed, Abdul Hafid Bajamal, Christianto Benjamin Lumenta

**Affiliations:** 1 Department of Neurosurgery, Faculty of Medicine, Universitas Airlangga, Dr. Soetomo General Hospital, Surabaya Neuroscience Institute, Surabaya, Indonesia; 2 Cell and Tissue Bank, Dr. Soetomo General Hospital, Surabaya, Indonesia; 3 Stem Cell Research and Development Center, Universitas Airlangga, Surabaya, Indonesia; 4 Department of Neurology, Faculty of Medicine, Universitas Airlangga, Dr. Soetomo General Hospital, Surabaya, Indonesia; 5 Department of Neurosurgery, Academic Teaching Hospital Munich-Bogenhausen, Technical University of Munich, Germany

**Keywords:** Haemorrhagic stroke, Bone marrow mesenchymal stem cells, Intraventricular transplantation, Outcome, NIHSS

## Abstract

**Object:**

Stroke, one of the most devastating diseases, is a leading cause of death and disability throughout the world and is also associated with emotional and economic problems. The main goal of this study was to investigate the clinical outcome of the intraventricular transplantation of bone marrow mesenchymal stem cells (BM-MSCs) in post-haemorrhagic stroke patients.

**Method:**

This study was done consisting of eight patients with supratentorial haemorrhagic stroke, who had undergone 24 weeks of standard treatment of stroke with stable neurological deficits. All of the patients received stem cell transplantation intraventricularly using autologous BM-MSCs. Six months and Twelve months after stem cells treatment, the clinical outcomes were measured using the National Institute of Health Stroke Scale (NIHSS) and adverse effect also observed.

**Result:**

The results of this study showed improvement of NIHSS score values before and after the treatment in five patients. No adverse effects or complications were detected during the 1-year observation.

**Conclusion:**

Intraventricular transplantation of BM-MSCs has shown benefits in improving the functional status of post-haemorrhagic stroke patients with no adverse effect.

## 1 INTRODUCTION

Stroke is one of the most devastating diseases and a leading cause of death and disability throughout the world [1]. Stroke is the world’s biggest fatal disease after ischaemic heart disease, accounting for a combined 15 million deaths in 2015. These diseases remained the leading causes of death globally from the last 15 years [2].

Intracerebral haemorrhagic stroke occurs in 10–15% of all strokes and has a higher mortality and disability rate than ischemic stroke or subarachnoid haemorrhage [3, 4, 5]. Spontaneous intracerebral haemorrhage (ICH) is classified into primary and secondary types according to cause [4]. Primary spontaneous ICH occurs in about 70–80% of cases and is caused by hypertension or amyloid angiopathy [4]. Secondary spontaneous ICH is often caused by vascular abnormalities, impaired blood clotting and coagulation, tumors and side effects of medications [3].

Brain tissue damage after ICH can be permanent [6]. Some mechanisms that can lead to neural damage include mass effects of blood clots to the surrounding tissue, inflammatory reactions, oxidative stress, neurotoxicity from excitatory neurotransmitters, thrombin and lysis of haemoglobin [3, 6, 7]. These mechanisms ultimately cause widespread cell death, leading to more severe damage after ICH [8]. Several neuroprotective agents have been developed to prevent such damage. Their mechanisms include suppressing the excitotoxicity of glutamate, inhibiting the inflammatory process, inducing antioxidant effects, suppressing the activity of matrix metalloproteinase (MMP) and preventing apoptosis [3, 9].

Although research and development into many therapeutic agents have been carried out to restore neural function after ICH, no optimal results have been obtained [6, 10]. Such therapies include, for example, neuroprotective drugs, hyperbaric therapy, hypothermia, hormonal therapy, and operative approaches with sophisticated and safe techniques. None of them has achieved the expected results, as the rates of mortality and disability by stroke remain high [6, 10].

In the last 10 years, alternative approaches to restoring neural function after stroke have been developed using the concept of neurorestoration by stem cell therapy [11]. Stem cell transplantation is expected to be a breakthrough in curing stroke patients, as it is expected to replace the damaged cells, improve the function of axons and restore the damaged neural circuitry [1, 3]. Additionally, stem cells could induce healing through the activation of endogenous neurogenesis, angiogenesis and synaptogenesis [3, 12]. Chopp *et al.* reported that stem cell therapy could prevent the occurrence of gliosis and the formation of scar tissue after stroke and, if given earlier, prevent apoptosis and suppress the inflammatory process [13].

The main goal of this study was to investigate the safety and feasibility of the intraventricular transplantation of bone marrow mesenchymal stem cells (BM-MSCs) in post-haemorrhagic stroke patients.

## 2 METHODOLOGY

This study received legal/ethical clearance from the local medical research ethics committees of Dr. Soetomo General Hospital, Surabaya, Indonesia, following the regulatory guidelines of the country. Moreover, a research permit was obtained from the Research and Development Department of East Java, Indonesia Provincial Government. Prior to the study, informed consent documents, details of the medical treatment and other necessary approval of the documents were delivered to all patients involved in the study.

The subjects of this study were supratentorial ICH patients with persistent neurological deficits after 24 weeks in Dr. Soetomo General Hospital, Surabaya, Indonesia.

Eight patients were included in the study. The clinical evaluation was carried out using the National Institute of Health Stroke Scale (NIHSS) 6 and 12 months after the first procedure of transplantation. The following inclusion and exclusion criteria were used for the patients.

### 2.1 Inclusion Criteria

- Haemorrhagic stroke patients
- Aged 45 to 65
- Supratentorial location
- No ventricular-peritoneal shunt
- No active infection/disease
- No systemic malignancies
- 24 weeks after stroke with stable NIHSS score in last 12 weeks

### 2.2 Exclusion Criteria

- Significant neurological improvement after stroke
- Did not receive optimal standard therapy
- Severe comorbid factors: pneumonia, heart diseases, diabetes mellitus, psychiatric disorders, uncontrolled hypertension
- Epilepsy

## 3 PROCEDURE

Isolation and intraventricular transplantation of bone marrow mesenchymal stem cells (BM-MSCs) was performed in the operating room of Dr. Soetomo General Hospital, Surabaya.

Autologous bone marrow stromal cell isolation was performed under general anaesthesia, and aspiration of BM-MSCs on patient's own iliac bone marrow was performed with a sterile procedure.

Isolation of mononuclear (MN) cells from bone marrow samples was done using Histopaque-density centrifugation methods. Whole MN cells were obtained and then cultured in Dulbecco's Eagle medium with 10% foetal bovine serum (FBS) (Sigma Chemical) in plastic petri humidity conditions of 37°C with 5% CO^2^. Unattached cells were discarded, while attached cells were expanded. When confluent cells were achieved (referred to as passage 0), cells were cultured up to passage 3.

Characterisation of BM-MSCs was performed using immunocytochemistry techniques. Cells were characterised with markers of fluorescein isothiocyanate (FITC) anti-human CD90 antibody (BioLegend, USA), phycoerythrin (PE) anti-human CD29 antibody (BioLegend, USA), peridinin-chlorophyll-protein complex: cyanine 5.5 conjugate (PerCp/Cy5.5) anti-human CD34 and anti-human CD45 FITC.

### 3.1 Transplantation Techniques

The BM-MSCs were injected into the patients using their own cells after they suffered supratentorial ICH with persistent neurological deficits over 24 weeks in Dr. Soetomo General Hospital, Surabaya, Indonesia. Under general anaesthesia, patients were conditioned in a supine position. The hair was shaved just behind the right frontal hairline, and then the area was washed with antiseptic solution. A mark was then made on the right Kocher point. A 2.5-cm wide linear incision was made in layers through the periosteum.

The process was continued by creating a burr hole in the calvaria and a small dural incision. An Ommaya reservoir was inserted into the ventricle, and then a maximum of 5cc cerebrospinal fluid was slowly aspirated through the Ommaya reservoir with a wing needle. Then, the stem cells were transplanted with the same wing needle (2 x 10^6^ cells in 3cc normal saline) and then flushed with 2cc normal saline. The surgical wound was then sutured layer by layer.

For booster transplantation, the same procedures were performed without the open procedure or general anaesthesia one month after the first transplantation. Hair did not need to be shaved, disinfection with povidone-iodine was performed at the skin and BM-MSC injection was carried out with the same dose using wing needle no. 25 through the subcutaneous transplanted Ommaya reservoir Fig. (**1**). Booster transplantation was done twice at one-month intervals.

## 4 RESULTS

### 4.1 Patient Characteristics

The average age of patients in this study was 53.44 ± 6.02 years. The youngest patient was 45 years old, and the oldest was 63 years old. The average age of patients was 54.25 ± 6.50 years.

In terms of gender, six patients (75%) were male, and two patients (25%) were female. All the patients had standard treatment for haemorrhagic stroke including medical treatment and physical rehabilitation. Two of the patients had surgical treatment (craniotomy and clot removal).

In terms of the site of supratentorial bleeding, five patients (62.5%) had bleeding in the basal ganglia, one patient (12.5%) had it in the right parietal and one patient (12.5%) had it as a right parieto-occipital haemorrhage. Further details on patient characteristics and clinical outcome are shown in (Table **1**).

### 4.2 Clinical Results

From the eight patients followed for 12 months, 5 patients showed improvement in neurologic status and 3 patients showed no change. One patient showed a motor repair and 2 patients showed improvement in the level of conception. Two other patients showed improvement from ataxia and improvement of aphasia and dysarthria. For a safety result evaluation, we also observe the possibility of adverse effects such as the incidence of increased intracranial pressure, infection, seizures and signs of rejection. And the result, we do not get any sign of such adverse effects during observation.

## 5 DISCUSSION

### 5.1 Patient Characterise

In terms of gender, 75% (6 patients) of the patients were male and 25% (2 patients) were female. The fact that there were more male than female patients is consistent with previous reports that stated that men are at a higher risk of developing stroke than women [14]. It is estimated that the incidence of stroke in women is less than that in men, with a ratio of 1.3 : 1 [15]. This is because women have the hormone estrogen that plays a role in maintaining immunity to menopause and as a protection or protector in the process of atherosclerosis, but after the woman experienced menopause, the risk of stroke between men and women became the same [14]. In our data, we have one post-menopause women age 59 years-old that suffered stroke.

In terms of age, three patients (37.5%) were aged 51–60, three patients (37.5%) were 45–50, while two patients (25%) were over 60. It has been widely reported that stroke is now tending to occur in younger patients [8]. In 1994, 12.9% of strokes occurred between the ages of 20 and 55. Then, in 2005, the number increased to 18.6%. The same study stated that the average age of stroke patients decreased from 71 in 1994 to 69 in 2005 [16]. Our reported results from eight patients demonstrates that five patients showed improvement after 48 weeks evaluation. Three patients (patients 1, 4, and 6) were more to cognitive improvements, and the remainder was a motoric (patient 5) and postural stability (patient 8). There was only a minor change in the power of arm and leg muscles (subjective analysis). Overall, the treatments demonstrated a strong safety profile with few reported improvements after 48 weeks evaluation.

Intracerebral haemorrhagic stroke accounts for 10–15% of all strokes and has a higher mortality and disability rate than ischemic stroke or subarachnoid haemorrhage stroke [4]. ICH tends to occur in the basal ganglia (40–50%), lobar regions (20–50%), thalamus (10–15%), pons (5–12%) and cerebellum (5–10%) [17]. In this study, there were nine patients (50%) with bleeding in the basal ganglia and six patients (31.25%) with lobar haemorrhages. This data is consistent with a previous study that found that basal ganglia haemorrhages are a more common pathology. Bleeding in the basal ganglia is usually associated with hypertension, whereas lobar ICH is often found in older patients with pathological cerebral amyloid angiopathy (CAA) [18].

### 5.2 Autologous BM-MSCs

Various kinds of cell types, including neural stem cells and BM-MSCs, have been widely studied in animals with stroke [5]. BM-MSCs are reported as having the most numerous benefits and optimal therapeutic potential, and they can be taken from the patients themselves to prevent ethical problems, body rejection reactions and other complications [5, 9, 19, 20].

Kondziolka *et al.* reported the results of their research on 14 ischemic stroke patients with intraparenchymal transplantation of human allograft neuronal cells in the basal ganglia without complications after the procedure. In the procedure, all of the patients were given immunosuppressant drugs and anti-seizure medications [15]. Savitz *et al.* also reported five patients with basal ganglia infarctions who had received intraparenchymal transplantations of foetal porcine neuronal cells, an allograft from animals. This procedure resulted in a seizure in one patient, and three patients experienced a decrease in neurological conditions. The study was stopped because of ethical problems and complications [21].

In this study, BM-MSCs were taken from the patients themselves, known as autologous BM-MSCs. This makes it ethically possible to perform the procedures and prevents further complications associated with stem cell sources. It is expected that the body’s rejection reaction will be minimal and it will not be necessary to use immunosuppressant drugs. Epileptogenic effects exist usually due to the irritating mass effects on foreign objects in the brain tissue. This was avoided, because BM-MSCs were given through the intraventricular route, avoiding the irritating effects and permanent pressure on the brain tissue.

Many studies on using adult mesenchymal stem cells (MSCs) have been experimentally carried out with a wide range of disease models in animals [9]. According to a consensus of MSC transplantation, MSCs taken from the bone marrow can be used in the process of tissue repair not only to stimulate the processes of proliferation, migration and differentiation from endogenous progenitor cells in all tissues of the body but also to suppress inflammatory processes and immune reactions and prevent apoptosis [5, 9].

The dose for stem cell transplantation has also varied widely in previous studies [1, 15, 22]. There is currently no standardised dose for stem cell therapy associated with the route of administration and the type of disease [5]. Research comparing stem cell doses by Wang *et al.* showed that it is necessary to determine an optimal dose for therapeutic stem cell transplantation, and a larger dose does not necessarily ensure better results [5]. For example, an overly high dose in intraparenchymal transplantation can affect the nutrition of grafted cells and, if given intravascularly, cause micro-emboli and vessel occlusion [5].

In this study, we used the dose of 2 x 10^6^ BM-MSCs, a higher dose than used in previous studies, with the intraventricular route applied directly into the intracranial space. Unlike the intraparenchymal transplantation techniques that have been mentioned before, with this route, the dose adjustment is more flexible, because it can be controlled by reducing the ventricular fluid if necessary based on the transplant dose. The risk of increased intracranial pressure and mass effects of the body can also be avoided. In this study, the dose of 2 x 10^6^ BM-MSCs was administered in 3 ml of fluid to avoid highly concentrated doses and excess fluid volume. No complications, such as signs of increased intracranial pressure, infections or seizures, were observed.

Neurogenesis in the subventricular zone (SVZ) depends on the combined stimulus of intrinsic and extrinsic signals [23, 24]. The source of extrinsic signals could be locally in the SVZ or from another area [23]. Extrinsic signals from BM-MSCs through the fluid could stimulate the cilia of ventricle ependymal cells and thus promote the proliferative activity of cells in the SVZ. The quality of proliferation and the number of neural stem cells in the SVZ are influenced by the strength of the stimulus (*i.e.*, a dose-dependent response) [24]. This also affects the subsequent recovery process [24, 25]. A more optimal dose results in a more optimal quality of subsequent neurogenesis.

### 5.3 Intraventricular Transplantation

The ventricular system has thin walls composed of ependymal cells [23, 26]. The permeable properties of ependymal cells make it quite effective for the treatment of certain medicines, including stem cell therapy targeting the brain parenchyma [26]. On the lateral ventricle, the ventricular walls are surrounded by the SVZ, which continuously produces new neurons [27, 28].

The SVZ was first discovered in mice, then in larger mammals, and later, it was found in humans [23, 26]. The location of the neurogenic niche area is very close to the lateral ventricle, which explains why the administration of stem cells through the intraventricular route is an effective method for stem cell therapy in stroke. The lateral ventricles are easy to access, enabling direct stimulation of the SVZ.

Moreover, in essence, cerebrospinal fluid is the endogenous regulatory factor of neuronal differentiation in neural regeneration, where the plexus choroideus produces substances during brain development or the regeneration process after brain injury [16]. The occurrence of this endogenous neurogenesis has been widely reported in several studies [28].

Intraventricular transplantation of stem cells in haemorrhagic stroke patients has never been reported. Three previous reports related to the subject were case reports: two were children with post-hypoxic encephalopathy, while the other was a case of amyotrophic lateral sclerosis (ALS). Jozwiak *et al.* performed intraventricular transplantation using autologous umbilical cord stem cells on a 16-month-old child who had post-hypoxic encephalopathy with good clinical results [29]. Baek *et al.* performed intraventricular transplantation using the Ommaya reservoir in cases of ALS. There were no reported complications from the study [30].

### 5.4 Safety Results

This is the first report of repeated stem cell transplantation through the intraventricular route. The application of stem cell therapy for stroke is very rational, as the principle of this therapy is to replace and repair the damaged brain tissue [19]. Recently, a theory suggesting that adult neural stem cells could be stimulated either exogenously or endogenously was put forth [28, 31]. Some recent research studies on stem cell therapy in stroke suggest some important issues that need further research: 1) sources of optimal stem cells, 2) most effective delivery route, 3) most effective time, and 4) optimal dose [13].

The results of the clinical evaluation of the eight patients with treatment showed no decrease in neurological status and no complications associated with the actions and effects from stem cells. Five patients showed improvement on NIHSS with different types of neurologic status enhancement. Some possible side effects that could be observed after treatment are 1) increased intracranial pressure, 2) seizures, 3) infection and 4) rejection reaction by the body. However, this study demonstrated that this technique is safe and reported no complications. One other advantage, the presence of the reservoir, facilitates repeated injections when applying booster therapy.

### 5.5 Clinical Outcomes

NIHSS scores were used to evaluate the neurological status of the stroke patients, determine the appropriate treatment for the patients and predict the patients’ prognosis [32]. Determining the severity of stroke is very important in assessing the prognosis associated with the disease, including mortality, length of stay in hospital, progress of neurological deficit and functional recovery of stroke patients. A study by Williams *et al.* showed that the reliability of the NIHSS was almost perfect [33]. Examining the NIHSS scores obtained prospectively and retrospectively is an excellent method for analysing patients’ conditions, and no bias on the scale was found in the retrospective process, although there were some missing elements of physical examinations in the medical records [33].

Kondziolka *et al.* implanted cultured human neural cells by the stereotactic technique in ischemic stroke patients. At the 18-month and 24-month observations, there were some functional improvements in patients [15]. This finding is consistent with this study. When we compared the ‘before and after’ conditions in stem cell transplantation, there were functional improvements in some patients.

Bang *et al.* reported the transplantation of BM-MSCs intravenously in patients with stroke infarction evaluated at months 3, 6 and 12. The results showed a significant difference in NIHSS scores in treated patients compared with controls [11]. Bhasin *et al.* reported a significant difference among six stroke infarction patients compared with controls after the intravenous transplantation of BM-MSCs. Post-treatment clinical and radiological evaluation was performed after eight weeks [11]. In this study, the evaluation was conducted after 48 weeks. All of the previous reports were studies on stroke infarction, whereas in this study, we conducted the research on haemorrhagic type.

Compared to ischemic stroke and subarachnoid haemorrhage, intracerebral haemorrhagic stroke has the highest mortality rate, more severe complications, and poorer long-term prognosis [3]. Based on the pathology, the tissue damage is more severe compared with ischemic stroke [3]. In the chronic phase, permanent damage or encephalomalacia frequently occurs, which usually does not respond to treatment, except physical rehabilitation therapy [2]. Based on these conditions, stem cell transplantation tends to work through the paracrine effects of trophic factors as a neuroprotective and stimulator agent of neuronal plasticity to improve the neurological function [3]. In this study included cases of supratentorial hemorrhagic stroke, which after transplantation procedures performed, there is an improvement in neurological status in some patients.

## CONCLUSION

In this study, clinical evaluations conducted using NIHSS scores after 24 and 48 weeks of treatment showed an improvement without evidence of side effects or complications. Because of the small sample size and non-randomised trial performed in this study, we could not reach a definitive conclusion regarding the potential of intraventricular transplantation of BM-MSCs in chronic stroke. However, this small study shows that repeated intraventricular transplantation of autologous BM-MSCs is advantageous. More advanced study is required to evaluate the efficacy of intraventricular stem cell therapy in stroke.

## Figures and Tables

**Fig. (1):**
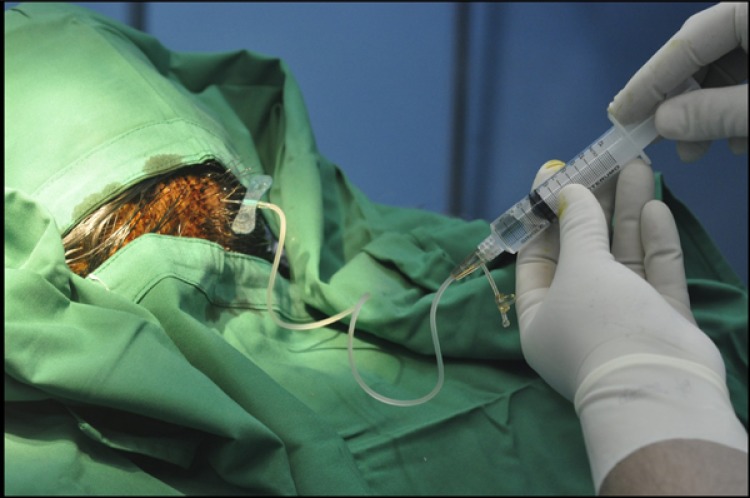
Booster transplantation is done by injection of BM-MSC through subcutaneous transplanted ommaya reservoir.

**Table 1:** Data progress of post-haemorrhagic stroke and post-treatment patients.

**No.**	**Gender (M/F)**	**Age** **(years old)**	**Location of Haemorrhage**	**Baseline**	**24 Weeks After Stroke**	**48-Weeks Evaluation**	Improvement
					**NIHSS**	**NIHSS**	
1.	F	45	Left Basal Ganglia	8	8	6	• Severe aphasia → Mild-to-moderate aphasia • Severe dysarthria → Mild-to-moderate dysarthria
2.	M	63	Right Parietal	2	2	2	-
3.	M	50	Left Basal Ganglia	5	5	5	-
4.	M	61	Left Basal Ganglia	4	4	3	LOC Questions; Answer one question correctly → Answer both question correctly
5.	M	56	Left Frontoparietal FP	23	22	19	• Left & Right Arm, some effort against gravity → Drift • Left & Right Leg, some effort against gravity → Drift
6.	M	52	Right Basal Ganglia	17	17	15	• Level of consciousness; not alert level 2 → Level of consciousness, not alert level 1 • LOC Commands; Performs neither task correctly → Performs one task correctly
7.	F	59	Right Parieto-occipital	5	5	5	-
8.	M	48	Left Basal Ganglia	5	5	4	Limb Ataxia; present in one limb → absent

## LIST OF ABBREVIATIONS

ALS: Amyotrophic lateral sclerosis
BM-MSC: Bone marrow mesenchymal stem cells
CAA: Cerebral amyloid angiopathy
CD: Cluster of differentiation
FBS: Foetal bovine serum
FITC: Fluorescein isothiocyanate
ICH: Intracerebral haemorrhage
MMP: Metalloproteinase
MN: Mononuclear cells
MSC: Mesenchymal stem cells
NIHSS: National Institute of Health Stroke Scale
PE: Phycoerythrin
PerCp/Cy5.5: Peridinin-chlorophyll-protein Complex
SVZ: Subventricular zone
